# Telemedicine for Remote Surgical Guidance in Endoscopic Retrograde Cholangiopancreatography: Mixed Methods Study of Practitioner Attitudes

**DOI:** 10.2196/20692

**Published:** 2021-01-11

**Authors:** Hedvig Aminoff, Sebastiaan Meijer, Urban Arnelo, Susanne Frennert

**Affiliations:** 1 Biomedical Engineering and Health Systems KTH Royal Institute of Technology Stockholm Sweden; 2 Department of Surgical and Perioperative Sciences, Surgery Umeå University Umeå Sweden; 3 Division of Surgery, CLINTEC Karolinska Institutet Stockholm Sweden; 4 Department of Computer Science and Media Technology Internet of Things and People Research Center Malmö University Malmö Sweden

**Keywords:** telemedicine, telementoring, teleconsulting, technology acceptance model, professional users, specialties, surgical/education, attitude, clinical staff, surgery, framework, surgeon, user-centered, interview, survey

## Abstract

**Background:**

Telemedicine innovations are rarely adopted into routine health care, the reasons for which are not well understood. Teleguidance, a promising service for remote surgical guidance during endoscopic retrograde cholangiopancreatography (ERCP) was due to be scaled up, but there were concerns that user attitudes might influence adoption.

**Objective:**

Our objective was to gain a deeper understanding of ERCP practitioners’ attitudes toward teleguidance. These findings could inform the implementation process and future evaluations.

**Methods:**

We conducted semistructured interviews with ERCP staff about challenges during work and beliefs about teleguidance. Theoretical constructs from the technology acceptance model (TAM) guided the thematic analysis. Our findings became input to a 16-item questionnaire, investigating surgeons’ beliefs about teleguidance’s contribution to performance and factors that might interact with implementation.

**Results:**

Results from 20 interviews with ERCP staff from 5 hospitals were used to adapt a TAM questionnaire, exchanging the standard “Ease of Use” items for “Compatibility and Implementation Climate.” In total, 23 ERCP specialists from 15 ERCP clinics responded to the questionnaire: 9 novices (<500 ERCP procedures) and 14 experts (>500 ERCP procedures). The average agreement ratings for usefulness items were 64% (~9/14) among experts and 75% (~7/9) among novices. The average agreement ratings for compatibility items were somewhat lower (experts 64% [~9/14], novices 69% [~6/9]). The averages have been calculated from the sum of several items and therefore, they only approximate the actual values. While 11 of the 14 experts (79%) and 8 of the 9 novices (89%) agreed that teleguidance could improve overall quality and patient safety during ERCP procedures, only 8 of the 14 experts (57%) and 6 of the 9 novices (67%) agreed that teleguidance would not create new patient safety risks. Only 5 of the 14 experts (36%) and 3 of the 9 novices (33%) were convinced that video and image transmission would function well. Similarly, only 6 of the 14 experts (43%) and 6 of the 9 novices (67%) agreed that administration would work smoothly. There were no statistically significant differences between the experts and novices on any of the 16 items (*P*<.05).

**Conclusions:**

Both novices and experts in ERCP procedures had concerns that teleguidance might disrupt existing work practices. However, novices were generally more positive toward teleguidance than experts, especially with regard to the possibility of developing technical skills and work practices. While newly trained specialists were the main target for teleguidance, the experts were also intended users. As experts are more likely to be key decision makers, their attitudes may have a greater relative impact on adoption. We present suggestions to address these concerns. We conclude that using the TAM as a conceptual framework can support user-centered inquiry into telemedicine design and implementation by connecting qualitative findings to well-known analytical themes.

## Introduction

### Background

Rapid development of surgical techniques and medical technology creates a continual need for retraining among surgeons [1,2]. Remote surgical guidance through telementoring and teleconsulting [3] can be a cost-effective way to facilitate teaching and training for less experienced surgeons [4] and support safe adoption of new clinical methods among experienced practitioners [5-9]. However, telemedicine innovations rarely move from the pilot stage to routine delivery [10,11]. As of yet, the factors contributing to acceptance and adoption of telemedicine are not well understood [12-15].

This study focuses on a promising telemedicine service for remote surgical guidance called teleguidance. The innovation was based on videoconferencing combined with transmission of high-quality endoscopic video and fluoroscopy. In this way, a high-volume clinical center could provide intraoperative consultation to a low-volume center during endoscopic retrograde cholangiopancreatography (ERCP), which is a highly specialized procedure for the diagnosis and treatment of biliary and pancreatic disease. A feasibility study demonstrated the impact on the clinical outcomes [16]. However, when teleguidance was to be scaled up to include more hospitals, some practitioners appeared less interested than anticipated. This raised concerns about implementation and about whether teleguidance could become an accepted way of working. We therefore conducted a theory-driven, user-centered study to gain a deeper understanding of practitioners’ attitudes toward teleguidance.

In the following sections, the clinical procedure and the telemedicine innovation are described. These sections also provide a general background to studying attitudes toward new technologies and a description of our methodological approach.

### Teleguidance in ERCP

ERCP is a technically advanced procedure for the diagnosis and treatment of biliary and pancreatic disease. ERCP has a long learning curve in both technical skills and decision making. After the initial specialty training, it is necessary to perform a certain number of cases per year to sustain the acquired skills, which may be difficult in low-volume clinics. Continual retraining, necessary for keeping up with new surgical advances [17], is also sometimes difficult at hospitals with fewer resources for education and research. This can have consequences in the case of unusual conditions or if complications arise during the procedure. Difficulties during ERCP can lead to delays in diagnosis and treatment or painful or even life-threatening complications for patients who already have serious underlying health issues [18].

**Figure 1:**
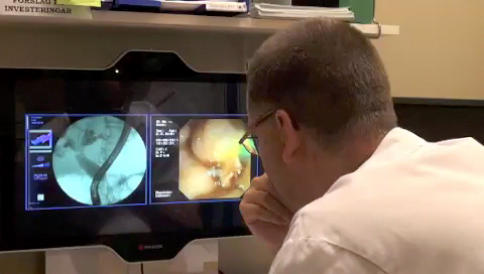
Teleguidance in endoscopic retrograde cholangiopancreatography.

Practitioners in need of advice during an ERCP procedure, but without the option to ask an experienced colleague on site, can opt for alternative procedures or refer the patient to another hospital. Another option is to get in touch with fellow specialists by telephone. Teleguidance was developed to enhance this practice through videoconferencing coupled with high-quality video transmission of videos and radiographic imagery (Figure 1).

A feasibility study has shown that teleguidance between a tertiary referral center and a low-volume hospital can result in improved quality of care [16]. This raised the prospect of extending this telemedicine service. A series of user-centered design efforts were initiated [19,20] as well as health-economic modeling [4]. Prior to expanding the service, an exploratory survey (Multimedia Appendix 1) showed variations in how clinicians rated their need of support. Subsequently, we wished to investigate practitioners’ attitudes toward teleguidance more thoroughly.

### Related Research

Traditionally, advanced surgical skills are learned by working together with experienced surgeons as mentors, progressing from shadowing to increasingly independent work. Necessary on-site training and retraining is sometimes difficult due to practical reasons and costs [9]. Numerous case studies—the earliest dating from the 1960s—have presented videoconferencing as a safe and efficacious way of providing surgical mentoring, enabling safe adoption of new techniques through remote expert guidance [2,6,7,21]. However, surgical telementoring is not widely used [5] and its impact over time is not well understood [6]. Implementation outcomes in health care are strongly affected by organizational context and how well an innovation answers to user needs [22]. This complexity makes it challenging to identify measurable determinants that provide an adequate image of implementation [23], in particular, regarding the quality and safety of products and services that function across multiple organizations [24].

Technology acceptance, a concept that relates beliefs and attitudes to use, is often considered an important determinant for technology implementation [22]. The technology acceptance model (TAM) [25] hypothesizes that people are more likely to use a technology if they believe it will be useful and easy to use. TAM was developed to provide validated measures for efficient early acceptance testing during development of office information systems [25,26]. The model defines two fundamental constructs: Perceived Usefulness signifying “the degree to which a person believes that using a particular system would enhance his or her job performance” and Perceived Ease of Use, representing “the degree to which a person believes that using a particular system would be free from effort” [27]. These central constructs were to be measured by a parsimonious questionnaire and were also intended to be transferable across different technologies and users [27]. TAM has been applied in many domains and TAM research has provided validation, extension, and elaboration of the central constructs [28]. However, surprisingly, few studies have investigated whether the model actually is a reliable predictor of use [28-31] or what makes a system useful [32]. Despite these weaknesses, the model is so frequently used that it has been described as a paradigm [30,33,34].

Various versions of TAM are commonly used in health care, and TAM has been extensively applied in studies of telemedicine [14,35-39]. While physician acceptance is commonly considered an important success factor [40], there does not appear to be any “optimal” version of TAM for telemedicine [14]. Despite its frequent use, TAM has shown shortcomings in health care [14,33,35]. Some of these have been attributed to the model’s narrow focus on individual users’ needs [39].

Another critique is that TAM invites quantitative treatment of narrowly defined theoretical constructs: the constructs themselves are treated as “black boxes,” which in the end has led to a state of theoretical confusion and chaos “around the TAM’s contribution to the understanding of technology acceptance and use” [32]. TAM was developed for prototype usability testing or system selection for office information technology systems, and the original definitions are grounded in research about cognitive and affective factors affecting the use of single-user computer software [25]. Transferring TAM to health care raises fundamental issues about how the carefully designed and validated TAM scale items (Multimedia Appendix 2) match the concepts being studied [41]. Holden et al [41] posit that acceptance studies in health care could benefit from a broad set of perceptions about usefulness and adapting the variables to the context in question. Many studies have added extensions to include a wider range of situational and social influences than were originally defined [14], and alternative conceptualizations of “Ease of Use” for health care broaden the focus from individual users’ beliefs about usability issues to include an organizational context [39].

The many adaptations and variants of TAM and the discussions about its relevance in health care highlight the importance of carefully considering what “Usefulness” and “Ease of Use” mean in each specific case. However, the model’s construct definitions can support qualitative data collection, analyses, and the interpretation of findings [42,43].

The combination of ambivalent attitudes toward teleguidance and research showing that telemedicine adoption appears problematic motivated us to investigate ERCP practitioners’ attitudes toward teleguidance. Guided by TAM, we studied the ways in which teleguidance might be perceived as “useful” and “easy to use” and how these perceptions vary across ERCP practitioners. These results are intended to inform the design and implementation process and to be valuable for understanding if and how teleguidance will be used at different clinical sites over time.

## Methods

### Questionnaire Design

Behavioral questionnaires should be grounded in the understandings of what is to be measured through contact with domain experts, and research in the relevant behavior domain should guide the construction of the items [44]. We conducted interviews to understand ERCP work and stakeholder beliefs about teleguidance’s contribution to procedures or other interactions with ERCP work. This was followed by thematic analysis [45], where TAM served as a theoretical framework. The interview findings served as a basis for adapting the standard TAM questionnaire. The interviews and questionnaire are described in more detail in the following sections.

### Interview Procedure

Initial key contacts with clinical staff at the different locations were set up by senior physicians at the tertiary referral center providing teleguidance, and a snowballing technique [46] gave us access to additional respondents. A total of 20 semistructured interviews with 10 ERCP specialists, 5 ERCP assistants, 3 technical staff, and 2 administrative staff from 5 hospitals were conducted.

Prior to each interview, the background, design, and purpose of the study, as well as the implications of participation were explained and also presented in printed from in order to gain informed consent [47] (Multimedia Appendix 3). Each participant was given a verbal presentation of the telemedicine service and a printed presentation with text and images describing teleguidance. Interviews were audio recorded and transcribed verbatim and treated as realist accounts. The interview length ranged from 30 minutes to 2 hours.

Coding and analysis proceeded through several iterations of reading the interview transcripts and refining the coding and themes. After coding, the data extracts were collated to help review patterns and relationships. The thematic analysis focused on identifying issues that might affect the use of teleguidance. At the outset, a number of themes were defined from the TAM model: statements related to performance, productivity, and effectiveness were to be coded as “Usefulness” issues, and issues related to expected usability or design issues as “Ease of Use” issues.

### Questionnaire Procedure

The 16-item questionnaire used a 7-point Likert-type scale, with a midpoint alternative to respond as “neutral.” It was made accessible as a closed, web-based 1-page survey provided through a web-based survey service during a 6-week period. Email invitations were sent to 25 physicians regularly performing ERCP at 15 different ERCP clinics, providing a link, information about estimated time to complete the survey (5 minutes), and information about data management and analysis (Multimedia Appendix 4). The number of practicing ERCP specialists in Sweden is small, and we made an effort to reach as many specialists in the field as possible that we had not yet interviewed. Analytical themes were operationalized as questionnaire items (Multimedia Appendix 5). Questionnaire items and phrasing were reworked a number of times to provide a succinct format and secure a high response rate. The order of the questions was randomized to avoid order effects. A few questions were also negatively phrased. Subjects’ age, gender, and professional experience, and an option to add comments was included. The questionnaire was pilot tested [46] by 2 ERCP specialists at the University hospital. The results were treated with exploratory data analysis methods, and we created visual representations of the score distributions in the form of stacked columns (Multimedia Appendix 6 and Multimedia Appendix 7). To gain interpretability and improve the stability of the ratings, we dichotomized the Likert scale ratings [48] with a cut between disagreement and neutral (1,2,3,4) and agreement (5,6,7). We also ran a Mann-Whitney *U* test in SPSS (IBM Corp), a rank-based nonparametric test, to investigate differences in the attitude scores between experts and novices for each questionnaire item. For all tests, a *P* value less than .05 was considered statistically significant.

## Results

### Interview Results

The interviews gave us insight into practitioner beliefs about teleguidance’s possible contributions to performance and factors that might interact with implementation. Four analytical themes (Multimedia Appendix 8) were defined through an iterative process of reviewing the transcripts, codes, and themes [49]. As the interview study progressed, it became clear that the respondents were not mentioning standard “Ease of Use” factors related to usability issues or design. What we found instead was mention about how teleguidance might interact with the work system, workflow issues, patients’ and management’s attitudes, and whether the telemedicine service would cause practical/technical or administrative issues. Risk was an additional theme that emerged inductively from the data sets. On this basis, we replaced the concept “Perceived ease of use” with 2 concepts defined in the Consolidated Framework for Implementation Research (CFIR) [50]: compatibility and implementation climate. Compatibility refers to the fit between the innovation and the current work systems. Implementation climate is intended to reflect users’ beliefs about whether the use of teleguidance would be expected and supported among important stakeholders. The themes were used as the basis for the questionnaire design.

### Questionnaire Results

In this study, 25 ERCP specialists—14 experts (>500 procedures) and 9 novices (<500 procedures)—provided complete responses (100% completion rate); 2 respondents were removed as they had previously participated in the interviews. The perceived usefulness items and average dichotomized agreement scores are shown in Table 1.

**Table 1:** Dichotomized agreement scores of perceived usefulness by experts and novices in endoscopic retrograde cholangiopancreatography.

Perceived usefulness items		Experts (n=14), n (%)	Novices (n=9), n (%)
**Performance**			
	Teleguidance can be a way for me to improve my technical skills in ERCP^a^	9 (64)	7 (78)
	The ERCP that we perform are challenging enough for teleguidance to be of value	7 (50)	6 (67)
	Overall, teleguidance would be beneficial for the quality and patient safety of the ERCP that we perform	11 (79)	8 (89)
	Teleguidance would help to further develop the ERCP activities at this clinic	10 (71)	8 (89)
**Effectiveness and productivity**			
	Teleguidance would allow my patients to get the appropriate treatment faster	10 (71)	6 (67)
	Teleguidance can allow my patients to receive a better ERCP treatment	11 (79)	8 (89)
	Teleguidance would allow us to perform a greater number of ERCP procedures	8 (57)	4 (44)
	Teleguidance can help me get the most out of the time I set aside for ERCP	8 (57)	7 (78)

^a^ERCP: endoscopic retrograde cholangiopancreatography.

The average agreement ratings for usefulness items were 64% (~9/14) among experts and 75% (~7/9) among novices. The average agreement ratings for compatibility items were somewhat lower (experts 64% [~9/14], novices 69% [~6/9]). The averages were calculated from the sum of several items and therefore, they only approximate the actual values. Experts and novices tended to agree that teleguidance could contribute to better overall ERCP treatment for patients (11/14, 79% and 8/9, 89%; respectively) and improve quality and patient safety during ERCP procedures (11/14, 79% and 8/9, 89%; respectively). However, only 7 of the 14 experts (50%) thought that the procedures they performed were challenging enough for teleguidance to be of value, while 6 of the 9 novices (67%) agreed. The novices also agreed to a higher extent (7/9, 78%) than experts (8/14, 57%) that teleguidance could contribute to their effectiveness. Fewer experts (10/14, 71%) than novices (8/9, 89%) believed teleguidance could help develop ERCP activities at the clinic. Multimedia Appendix 6 shows the score distributions of the Usefulness items.

Experts gave high agreement scores (>75%) on both implementation climate items (Table 2), while relatively few novices agreed that management would be positive toward teleguidance (experts 11/14, 79%; novices 7/9, 56%). There were also concerns about the quality of video and image transmission and administration between hospitals, with relatively low agreements on “The quality of video and image transmission between hospitals will function well” (experts 5/14, 36%; novices 3/9, 33%) and “Administration between hospitals will function well” (experts 6/14, 43%; novices 6/9, 67%). Similarly, only 8 of the 14 experts (57%) and 6 of the 9 novices (67%) agreed that teleguidance would not create new patient safety risks. This contrasts with the scores for “teleguidance would be beneficial for the quality and patient safety of the ERCP that we perform,” where both groups expressed positive expectations about the service’s contribution to quality and patient safety.

**Table 2:** Dichotomized agreement scores on implementation climate and compatibility by experts and novices in endoscopic retrograde cholangiopancreatography.

Perceived ease of use items		Experts (n=14), n (%)	Novices (n=9), n (%)
**Implementation climate**			
	Management would be positive that I use teleguidance	11 (79)	5 (56)
	My patients would be positive that I use teleguidance	11 (79)	7 (78)
**Compatibility**			
	Teleguidance is a way of working that could suit me and my workplace	10 (71)	9 (100)
	The quality of video and image transmission between hospitals will function well	5 (36)	3 (33)
	Administration between hospitals will function well	6 (43)	6 (67)
	Teleguidance is unlikely to create risks for patients' confidentiality and integrity	10 (71)	7 (78)
	Teleguidance is unlikely to create risks for staff integrity	11 (79)	7 (78)
	Teleguidance is unlikely to create new patient safety risks	8 (57)	6 (67)

The graphs showing score distributions (Multimedia Appendix 7) illustrate that agreement was generally high; however, there was a large portion of neutral ratings on items, namely, “Management would be positive that I use teleguidance,” “The quality of video and image transmission between hospitals will function well,” and “Administration between hospitals will function well.”

Mann-Whitney *U* tests showed that there were no statistically significant differences between the novices and the experts on any of the 16 items (*P*<.05) (Multimedia Appendix 9).

## Discussion

### Principal Results

The interviews provided insight into the types of benefits teleguidance could provide and into the everyday ERCP work practices that might be affected by teleguidance. This served as important input for our questionnaire, where we defined the construct “Usefulness” in terms of how teleguidance might contribute to performance, productivity, and effectiveness. We exchanged the standard TAM construct “Ease of Use” for “Implementation climate and Compatibility” to better reflect concerns about how teleguidance might fit with the existing work system and if teleguidance might introduce new risks. Our main focus was to develop an understanding of the complexity of the domain and of the diversity among respondents, grounded in qualitative data.

The questionnaire allowed us to expand our inquiry to include a larger number of specialists in a domain where access to practitioners can be very difficult [51]. In the interviews, many expressed positive expectations about teleguidance, particularly that it could answer to challenges that novices were facing. However, many staff members also expressed concerns about how teleguidance would fit in with existing work system. The questionnaire results similarly showed that most respondents believed that teleguidance could contribute to the quality and safety of procedures and to the many anticipated technical and administrative issues—possibly even new patient safety issues. This indicates that practitioners had concerns that teleguidance might disrupt work.

While novices were the primary target group of the telemedicine service, teleguidance was designed with both novices and more experienced ERCP specialists in mind. We found that some experts were consistently skeptical toward teleguidance. As senior clinicians are more likely to be key decision makers [52], the attitudes among this group can have a greater relative importance for implementation than novices’ attitudes. Below, we discuss some possible reasons for and consequences of the differences between novices and experts and comment on the methodological concerns. We conclude with some practical suggestions for the implementation of teleguidance.

### Differences Between Novices and Experts

Our interviews indicated that novices could be under considerable pressure during key phases of the procedure and they often saw room for improvement in current work processes. This was reflected in the questionnaire results, where novices had higher agreement scores on all the performance items and on the items for individual and organizational effectiveness. Novices may also have a lower threshold to work with videoconferencing than their more experienced colleagues, who also were older; previous use of information and communication technology in everyday life has been seen as a significant predictor for physicians’ telemedicine use [53]. The score distributions show that there were some items with many neutral responses, especially the “Implementation climate and Compatibility” items. This not only draws down the dichotomized score but it also indicates a challenge in asking potential users to form an opinion of a complex intervention, which might have complex outcomes, eg, patient safety issues. Experienced practitioners displayed a lower level of agreement that teleguidance could improve their individual performance, which may be explained by a less imperative need for support. However, developing integrative competence and taking part in a surgical innovation is an important aspect of sustaining acquired surgical competence [54], which is one of the aims of teleguidance.

While more experts than novices believed that management would be positive toward teleguidance, they also expected more administrative challenges. The differences in how experts and novices weighed these organizational aspects of teleguidance may be explained both by differences in roles and in experience: senior practitioners were more likely to have managerial functions and hence might have a different perspective of management priorities and the changes that teleguidance might entail [2]. We can only speculate about the experts who gave negative ratings consistently: senior ERCP specialists with established practices and status may perceive teleguidance more as a challenge to traditional routines [55] or professional autonomy [56], and as a consequence, tend to prefer existing work practices [57]. However, it is likely that negative attitudes are a common source of bias in implementation studies, as these practitioners may very well decline to participate at all. Research has shown that differences in power and politics among professional groups influence the use of new technologies in health care [52]. Our findings underline the importance of including a variety of experience and roles in this type of study: some experts thought that novices would prefer “hands-on” help to remote guidance, while many novices themselves said the opposite. In addition, many of the interviewed nurses mentioned concerns about staff and patient integrity issues, but nurses were not included in the questionnaire nor was this view reflected in the doctors’ questionnaire responses.

### Practical Implications for Implementation

Teleguidance was initially developed to meet a wide range of challenges regarding the quality of ERCP procedures; it was not exclusively intended to serve the practical training needs of novices. Negative attitudes, even among a smaller group of experienced practitioners, may offset implementation efforts. However, our findings provide some guidance for design and implementation. The interviews provided insight into ERCP as a time-sensitive, collaborative team effort, highly dependent on medical technologies. Staff concerns that teleguidance might be an extra burden is based on daily experiences of ERCP work. Viewing teleguidance as a service rather than a new technology can widen the design perspective to include considerations what happens when two work systems are bridged by telemedicine. Experts’ concerns about administrative issues and compatibility of work practices should be taken seriously; implementation efforts could benefit from identifying workflow issues, defining staff roles and tasks, and designating scheduling allowances for teleguidance-related tasks. If teleguidance is to be widely used, it is important to define and communicate the value of teleguidance even for experienced practitioners and to investigate incentives for experts’ participation, since teleguidance also aims to support learning among experts. The SAGES telementoring initiative [2] differentiates between telementoring and teleconsultation, answering to different needs among experts and novices. Their definition of telementoring emphasizes a learning relationship between a mentor and a mentee and that telementoring occurs within an educational framework. Framing teleguidance in a similar way could benefit all parties; by clarifying relationships and objectives, teleguidance may be implemented as an explicit training effort for novices. Well-defined educational objectives might serve as incentives for novices to participate as well as increase the management support of telementoring. This could be a way to avoid inadvertently challenging the power and autonomy of incumbent experts.

As a contrast, teleguidance between two qualified experts might be defined as teleconsultation. This would signify different content and implications of the practice, with an emphasis on an exchange between peers, which may be experienced as less of a threat or intrusion by the more experienced ERCP specialists.

### Limitations

This study has limitations due to the lack of lack of internal validity tests, which were beyond the scope of the study. This study does not attempt to exhaustively identify themes that may affect attitudes toward teleguidance, as the TAM guided toward predefined factors of interest. The number of respondents may be questioned, but as the total population of practicing ERCP specialists in Sweden is small and our respondents are highly representative, we claim to adequately cover variations among the groups. This study was exploratory, focused on developing an understanding of the complexity of the domain and of diversity among respondents. The quality of our findings is grounded in the qualitative data, rather than in statistical inference [58]. This study represents “the scientific discovery phase” [42], where empirical findings from a complex setting can ground hypotheses about behavior and design. In this sense, items with low agreement or ambiguous findings such as the seemingly contradictory beliefs about patient safety are valuable indications about how similar studies can be refined.

### Conclusion

In our interviews, practitioners’ descriptions of ERCP work and their beliefs about teleguidance did not resonate with the classical TAM questionnaire; they had no need to “work more quickly” or “make the job easier” nor did the interviews provide any statements about “Ease of Use” issues such as design features or usability. Instead, staff spoke of organizational demands deeply infused with clinical work, intense team collaboration, and constant organizational pressure for effectiveness and efficiency.

This means that teleguidance does not just have to answer to individual users’ needs but also to organizational demands and priorities. Our findings show that introducing teleguidance is not “just” introducing new technology; teleguidance will change collaborative practices, linking locations that have their own sets of practices and priorities, which also can cause disruptions. We believe that the main cause for negative attitudes toward teleguidance is based on these concerns, which can be addressed during design and implementation. This study is an example of how TAM can support theory-guided user-centered design approaches to telemedicine development [31]. This may be a way to tackle the complexity of introducing technology in health care [59]. Using TAM in this way is also a return to the original intentions of the TAM, namely, to provide early user feedback to the system development processes, so as to gain better understanding of “how to improve user acceptance through design” [26].

### Future Work

We suggest that using theories to guide the investigation of relevant user needs and expectations in a specific context is a way to inform the development and implementation of telemedicine. By connecting findings to well-known analytical themes such as usefulness and terminology and concepts in frameworks such as CFIR [50], this type of qualitative approach can contribute to understanding the forces that shape the adoption of telemedicine and contribute to its effects. The complexity of introducing teleguidance across multiple clinical sites and ERCP teams will make evaluation particularly challenging [60,61]. Theories that accommodate complexity in studies of technological change are increasingly emphasized [59,62]. Building on our insights from this study, we hope to apply sociotechnical methods that are developed for understanding changes in complex and adaptive settings [59] and follow the introduction of teleguidance over time in a real-world context to study the ways in which teleguidance affects user behaviors.

## Appendix

Multimedia Appendix 1 Results of the exploratory needs survey.

Multimedia Appendix 2 Original technology acceptance model items (Davis, 1989).

Multimedia Appendix 3 Consent form for the interviews.

Multimedia Appendix 4 Invitation and information for participation in the questionnaire.

Multimedia Appendix 5 Operationalizations and questionnaire items.

Multimedia Appendix 6 Distribution of agreement scores on usefulness.

Multimedia Appendix 7 Distribution of agreement scores on implementation climate and compatibility.

Multimedia Appendix 8 Analytical themes and examples of coded content.

Multimedia Appendix 9 Mann-Whitney U test for comparison between novices and experts: scale 1 (strongly disagree) to 7 (strongly agree).

## Abbreviations

CFIR: consolidated framework for implementation research
ERCP: endoscopic retrograde cholangiopancreatography
TAM: technology acceptance model

## References

[1] No authors listed (1995). Statements on emerging surgical technologies and the evaluation of credentials. American College of Surgeons. Surg Endosc.

[2] Schlachta CM, Nguyen NT, Ponsky T, Dunkin B (2016). Project 6 Summit: SAGES telementoring initiative. Surg Endosc.

[3] Huang EY, Knight S, Guetter CR, Davis CH, Moller M, Slama E, Crandall M (2019). Telemedicine and telementoring in the surgical specialties: A narrative review. Am J Surg.

[4] Brinne Roos J, Bergenzaun P, Groth K, Lundell L, Arnelo U (2020). Telepresence-teleguidance to facilitate training and quality assurance in ERCP: a health economic modeling approach. Endosc Int Open.

[5] Augestad KM, Han H, Paige J, Ponsky T, Schlachta CM, Dunkin B, Mellinger J (2017). Educational implications for surgical telementoring: a current review with recommendations for future practice, policy, and research. Surg Endosc.

[6] Erridge S, Yeung DKT, Patel HRH, Purkayastha S (2019). Telementoring of Surgeons: A Systematic Review. Surg Innov.

[7] Shimizu S, Itaba S, Yada S, Takahata S, Nakashima N, Okamura K, Rerknimitr R, Akaraviputh T, Lu X, Tanaka M (2011). Significance of telemedicine for video image transmission of endoscopic retrograde cholangiopancreatography and endoscopic ultrasonography procedures. J Hepatobiliary Pancreat Sci.

[8] El-Sabawi B, Magee William (2016). The evolution of surgical telementoring: current applications and future directions. Ann Transl Med.

[9] Antoniou SA, Antoniou GA, Franzen J, Bollmann S, Koch OO, Pointner R, Granderath FA (2012). A comprehensive review of telementoring applications in laparoscopic general surgery. Surg Endosc.

[10] Zanaboni P, Wootton R (2012). Adoption of telemedicine: from pilot stage to routine delivery. BMC Med Inform Decis Mak.

[11] Standing C, Standing S, McDermott M, Gururajan R, Kiani Mavi R (2016). The Paradoxes of Telehealth: a Review of the Literature 2000-2015. Syst Res.

[12] Flodgren G, Rachas A, Farmer A, Inzitari M, Shepperd S (2015). Interactive telemedicine: effects on professional practice and health care outcomes. Cochrane Database Syst Rev.

[13] Ryu S (2012). Telemedicine: Opportunities and Developments in Member States: Report on the Second Global Survey on eHealth 2009 (Global Observatory for eHealth Series, Volume 2). Healthc Inform Res.

[14] Rahimi B, Nadri H, Lotfnezhad Afshar H, Timpka T (2018). A Systematic Review of the Technology Acceptance Model in Health Informatics. Appl Clin Inform.

[15] Augestad KM, Bellika JG, Budrionis A, Chomutare T, Lindsetmo R, Patel H, Delaney C, Mobile Medical Mentor (M3) Project (2013). Surgical telementoring in knowledge translation--clinical outcomes and educational benefits: a comprehensive review. Surg Innov.

[16] Påhlsson HI, Groth K, Permert J, Swahn F, Löhr M, Enochsson L, Lundell L, Arnelo U (2013). Telemedicine: an important aid to perform high-quality endoscopic retrograde cholangiopancreatography in low-volume centers. Endoscopy.

[17] Society of American GastrointestinalEndoscopic Surgeons (SAGES) Guidelines Committee (2007). Guidelines for training in diagnostic and therapeutic endoscopic retrograde cholangiopancreatography (ERCP). Surg Endosc.

[18] Olsson G, Arnelo U, Swahn F, Törnqvist Björn, Lundell L, Enochsson L (2017). The H.O.U.S.E. classification: a novel endoscopic retrograde cholangiopancreatography (ERCP) complexity grading scale. BMC Gastroenterol.

[19] Groth K, Arnelo U, Bergenzaun P, Lundell L, Aminoff H, Frykholm O, Sundkvist A (2016). Designing for interactivity in a tele-guidance setting. Int J Integr Care.

[20] Frykholm Oscar, Aminoff Hedvig, Groth Kristina, Arnelo Urban (2015). User-centered design of ERCP teleguidance.

[21] Lee SP, Lee HL, Hahm JS, Choi HS, Joe I, Shimizu S (2012). International live endoscopic multichannel demonstration using superfast broadband internet connections. Clin Endosc.

[22] Greenhalgh T, Swinglehurst D, Stones R (2014). Rethinking resistance to ‘big IT’: a sociological study of why and when healthcare staff do not use nationally mandated information and communication technologies. HEALTH SERVICES AND DELIVERY RESEARCH.

[23] Plsek PE, Greenhalgh T (2001). Complexity science: The challenge of complexity in health care. BMJ.

[24] Carayon P (2006). Human factors of complex sociotechnical systems. Appl Ergon.

[25] Davis FD (1989). Perceived Usefulness, Perceived Ease of Use, and User Acceptance of Information Technology. MIS Quarterly.

[26] Davis FD (1993). User acceptance of information technology: system characteristics, user perceptions and behavioral impacts. International Journal of Man-Machine Studies.

[27] Davis FD, Bagozzi RP, Warshaw PR (1989). User Acceptance of Computer Technology: A Comparison of Two Theoretical Models. Management Science.

[28] Lee Y, Kozar KA, Larsen KR (2003). The Technology Acceptance Model: Past, Present, and Future. CAIS.

[29] Legris P, Ingham J, Collerette P (2003). Why do people use information technology? A critical review of the technology acceptance model. Information & Management.

[30] Turner M, Kitchenham B, Brereton P, Charters S, Budgen D (2010). Does the technology acceptance model predict actual use? A systematic literature review. Information and Software Technology.

[31] Harst L, Lantzsch H, Scheibe M (2019). Theories Predicting End-User Acceptance of Telemedicine Use: Systematic Review. J Med Internet Res.

[32] Benbasat I, Barki H (2007). Quo vadis TAM?. JAIS.

[33] Holden RJ, Karsh B (2010). The technology acceptance model: its past and its future in health care. J Biomed Inform.

[34] Bagozzi R (2007). The Legacy of the Technology Acceptance Model and a Proposal for a Paradigm Shift. JAIS.

[35] Ammenwerth E (2019). Technology Acceptance Models in Health Informatics: TAM and UTAUT. Stud Health Technol Inform.

[36] Hayotte M, Thérouanne Pierre, Gray L, Corrion K, d'Arripe-Longueville F (2020). The French eHealth Acceptability Scale Using the Unified Theory of Acceptance and Use of Technology 2 Model: Instrument Validation Study. J Med Internet Res.

[37] Safi S, Danzer G, Schmailzl KJ (2019). Empirical Research on Acceptance of Digital Technologies in Medicine Among Patients and Healthy Users: Questionnaire Study. JMIR Hum Factors.

[38] Schweitzer M, Huber L, Gorfer T, Hörbst A (2020). Experiences With Developing and Using Vital Sign Telemonitoring to Support Mobile Nursing in Rural Regions: Feasibility and Usability Study. JMIR Nursing.

[39] Jacob C, Sanchez-Vazquez A, Ivory C (2020). Social, Organizational, and Technological Factors Impacting Clinicians' Adoption of Mobile Health Tools: Systematic Literature Review. JMIR Mhealth Uhealth.

[40] Wade VA, Eliott JA, Hiller JE (2014). Clinician acceptance is the key factor for sustainable telehealth services. Qual Health Res.

[41] Holden RJ, Brown RL, Scanlon MC, Karsh B (2012). Modeling nurses' acceptance of bar coded medication administration technology at a pediatric hospital. J Am Med Inform Assoc.

[42] Roth E, Patterson E, Montgomery Henry, Lipshitz Ranaan, Brehmer Berndt (2005). Using observational study as a tool for discovery: Uncovering cognitive and collaborative demands and adaptive strategies. How Professionals Make Decisions.

[43] Roth EM (2008). Uncovering the Requirements of Cognitive Work. Hum Factors.

[44] Anastasi A (1986). Evolving Concepts of Test Validation. Annu Rev Psychol.

[45] Pettigrew AM (1990). Longitudinal Field Research on Change: Theory and Practice. Organization Science.

[46] Breakwell G, Hammond S, Fife-Schaw C (2002). Surveys and sampling issues. Research methods in psychology.

[47] Arksey H, Knight P, Arksey H, Knight P (2012). Protecting Rights and Welfare. Interviewing for social scientists: An introductory resource with examples.

[48] Merkys G, Bubeliene D, Agarwal N, Sakalauskas L, Weber GW (2019). Optimization of Data Processing and Presentation in Social Surveys: From Likert-Means to “Yes Percentage”. Modeling and Simulation of Social-Behavioral Phenomena in Creative Societies.

[49] Braun V, Clarke V (2006). Using thematic analysis in psychology. Qualitative Research in Psychology.

[50] Damschroder LJ, Aron DC, Keith RE, Kirsh SR, Alexander JA, Lowery JC (2009). Fostering implementation of health services research findings into practice: a consolidated framework for advancing implementation science. Implement Sci.

[51] Furniss D, Randell R, O’Kane Aa, Taneva S, Mentis H, Blandford A, Furniss D, Randell R, O'Kane A, Taneva S (2014). Fieldwork for Healthcare: Guidance for Investigating Human Factors in Computing Systems. Synthesis Lectures on Assistive, Rehabilitative, and Health-Preserving Technologies.

[52] Robert G, Greenhalgh T, MacFarlane F, Peacock R (2010). Adopting and assimilating new non-pharmaceutical technologies into health care: a systematic review. J Health Serv Res Policy.

[53] Lacasta Tintorer D, Flayeh Beneyto S, Manresa JM, Torán-Monserrat Pere, Jiménez-Zarco Ana, Torrent-Sellens J, Saigí-Rubió Francesc (2015). Understanding the discriminant factors that influence the adoption and use of clinical communities of practice: the ECOPIH case. BMC Health Serv Res.

[54] Wani S, Keswani RN, Petersen B, Edmundowicz SA, Walsh CM, Huang C, Cohen J, Cote G (2018). Training in EUS and ERCP: standardizing methods to assess competence. Gastrointest Endosc.

[55] Chau PY, Hu PJ (2002). Investigating healthcare professionals’ decisions to accept telemedicine technology: an empirical test of competing theories. Information & Management.

[56] Walter Z, Lopez MS (2008). Physician acceptance of information technologies: Role of perceived threat to professional autonomy. Decision Support Systems.

[57] Kim HW, Kankanhalli A (2009). Investigating User Resistance to Information Systems Implementation: A Status Quo Bias Perspective. MIS Quarterly.

[58] Jansen H (2010). The Logic of Qualitative Survey Research and its Position in the Field of Social Research Methods. Forum: Qualitative Social Research.

[59] Greenhalgh T, Wherton J, Papoutsi C, Lynch J, Hughes G, A'Court Christine, Hinder S, Procter R, Shaw S (2018). Analysing the role of complexity in explaining the fortunes of technology programmes: empirical application of the NASSS framework. BMC Med.

[60] Woods D, Dekker S (2000). Anticipating the effects of technological change: A new era of dynamics for human factors. Theoretical Issues in Ergonomics Science.

[61] Greenhalgh T, Papoutsi C (2018). Studying complexity in health services research: desperately seeking an overdue paradigm shift. BMC Med.

[62] Moore GF, Audrey S, Barker M, Bond L, Bonell C, Hardeman W, Moore L, O'Cathain A, Tinati T, Wight D, Baird J (2015). Process evaluation of complex interventions: Medical Research Council guidance. BMJ.

