# Supplementary material for: Telemedicine for Remote Surgical Guidance in Endoscopic Retrograde Cholangiopancreatography: Mixed Methods Study of Practitioner Attitudes
Source: JMIR Form Res. 2021 Jan 11;5(1):e20692. doi: 10.2196/20692 (PMC7834938; doi:10.2196/20692)
Supplement: Multimedia Appendix 1 [file formative_v5i1e20692_app1.docx]

# Results: exploratory questionnaire “Interest for Teleguidance”

The exploratory questionnaire about the interest for teleguidance was distributed via paper case report forms to ERCP clinics at 9 hospitals. provided 271 responses from 9 different hospitals. 271 case report forms were returned, with an estimated response rate of 75% compared with data from the National Quality Registry for Gallstone Surgery and Endoscopic Retrograde Cholangiopancreatography (Gallriks)[1] for the corresponding period and hospitals.

In 21% (n = 56) of the ERCP surveys, respondents assessed that there was a need for Teleguidance and in 4% (n = 12) there was uncertainty about the need. There was a great variety between the estimated need at different hospitals (0-42%) and there was also variation in how much effect one believed teleguidance would have. Only 9 out of 56 who replied that they needed teleguidance believed it could have decisive meaning.

The most requested support was general assessment (37.5%) as well cannulation (28.6%), or a combination of several different considerations (16.1%).

Endoscopists believe that teleguidance could, among other things, prevent reERCP in 34% and re-operation, PTC or other interventions in 16%.

The cases where the need for teleguidance seems to be greatest was when respondents gave the indication "something else"; that the patient is not previously sphincterotomized or when experiencing that one completely or partially fails with the ERCP.

1. Enochsson L, Thulin A, Osterberg J, Sandblom G, Persson G. The Swedish Registry of Gallstone Surgery and Endoscopic Retrograde Cholangiopancreatography (GallRiks): A nationwide registry for quality assurance of gallstone surgery. JAMA Surg 2013;**148**(5):471-8 doi: 10.1001/jamasurg.2013.1221[published Online First: Epub Date]|.
