# Supplementary figures and images for: Telemedicine for Remote Surgical Guidance in Endoscopic Retrograde Cholangiopancreatography: Mixed Methods Study of Practitioner Attitudes
Source: JMIR Form Res. 2021 Jan 11;5(1):e20692. doi: 10.2196/20692 (PMC7834938; doi:10.2196/20692)

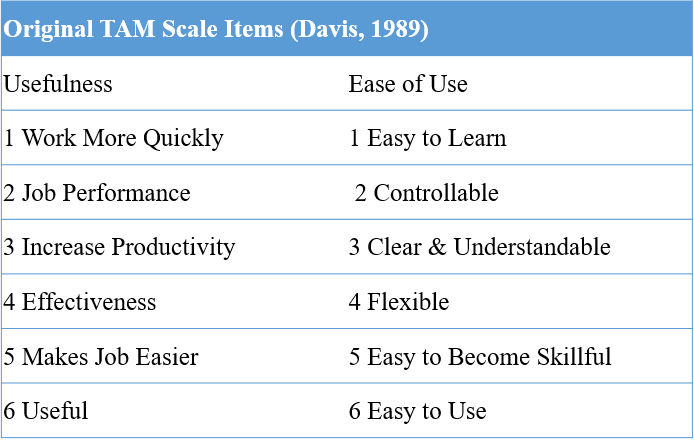

Supplement: Multimedia Appendix 2 [file formative_v5i1e20692_app2.png]

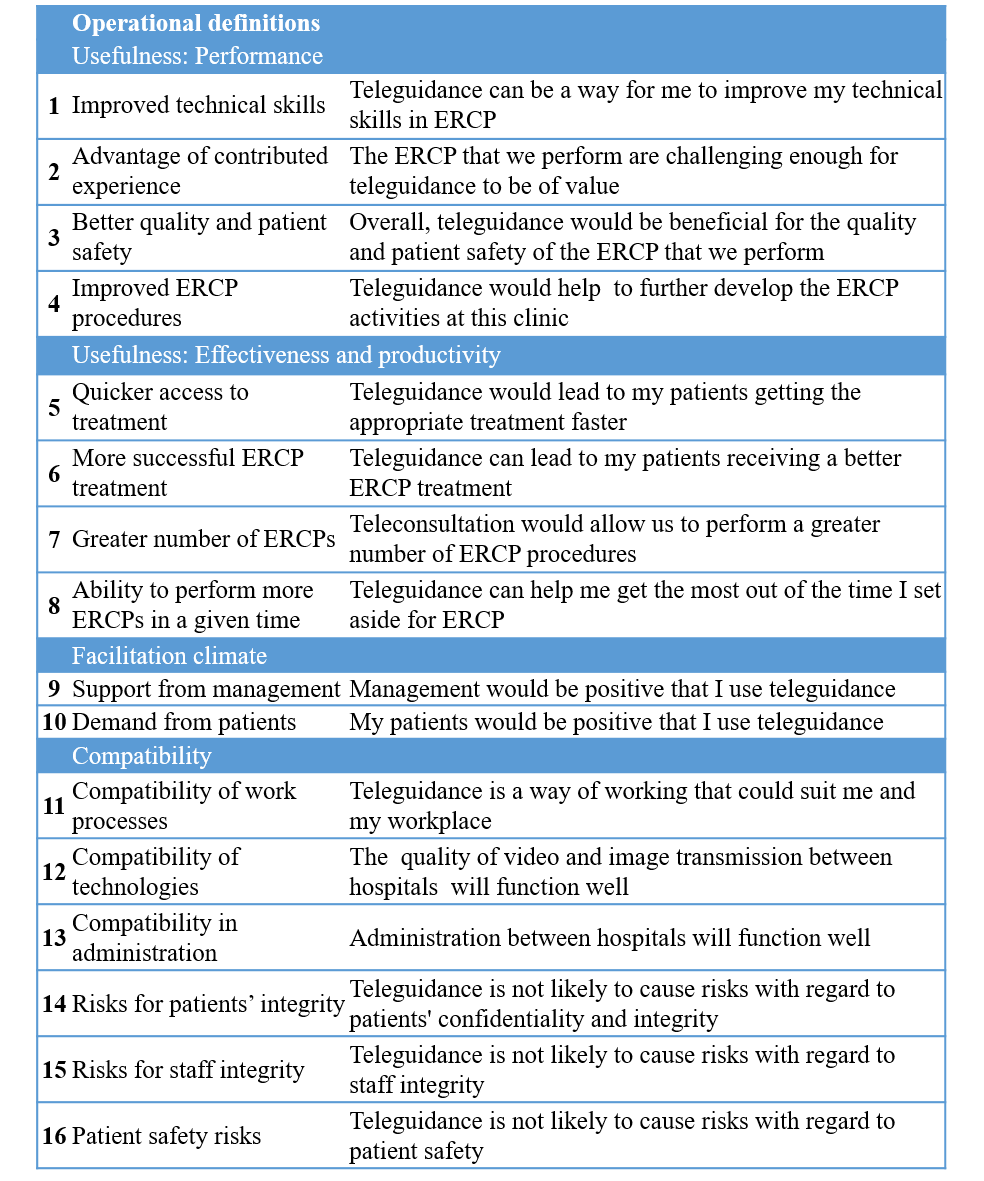

Supplement: Multimedia Appendix 5 [file formative_v5i1e20692_app5.png]

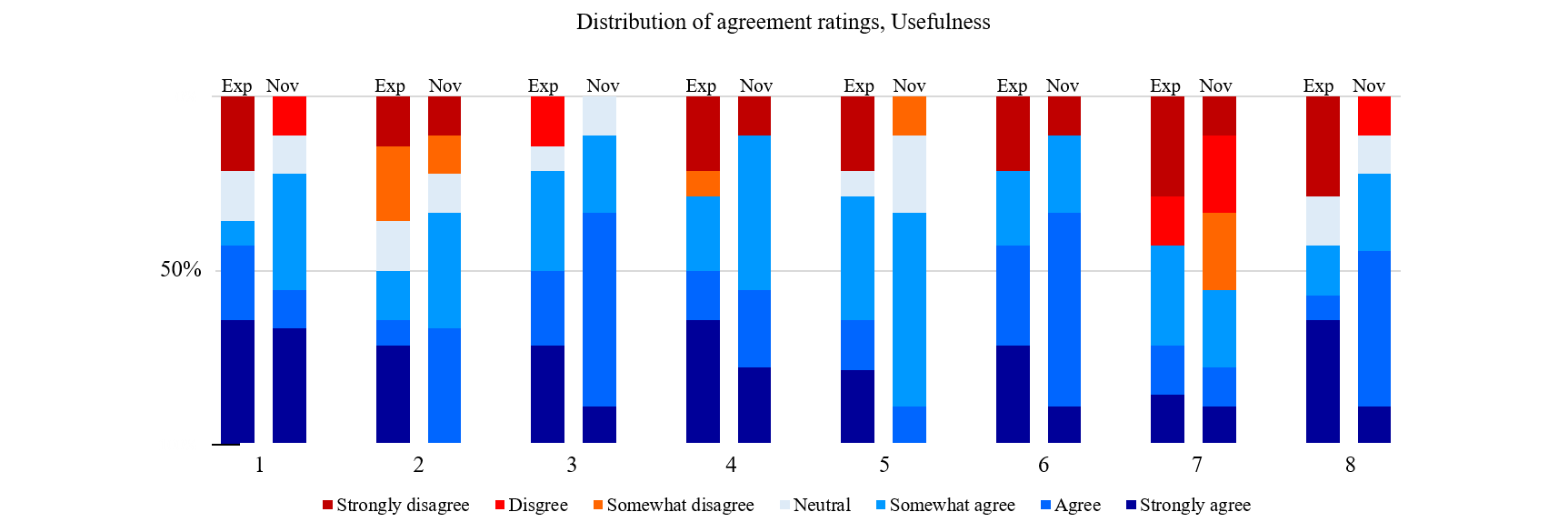

Supplement: Multimedia Appendix 6 [file formative_v5i1e20692_app6.png]

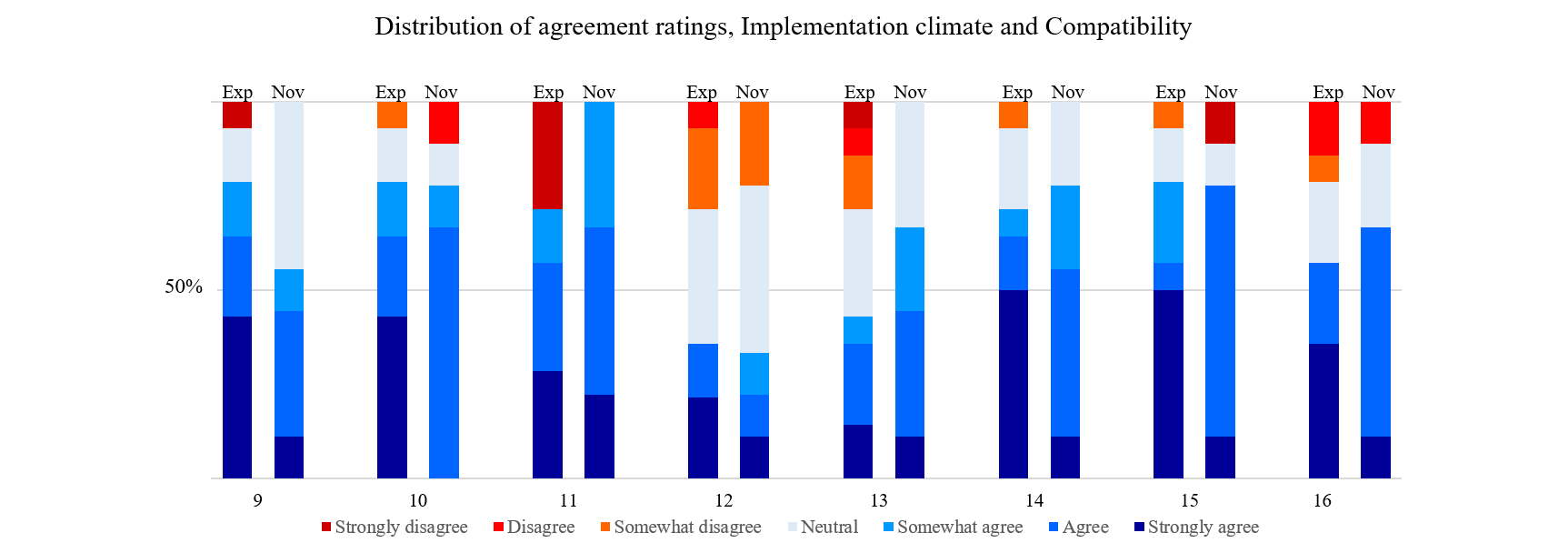

Supplement: Multimedia Appendix 7 [file formative_v5i1e20692_app7.png]

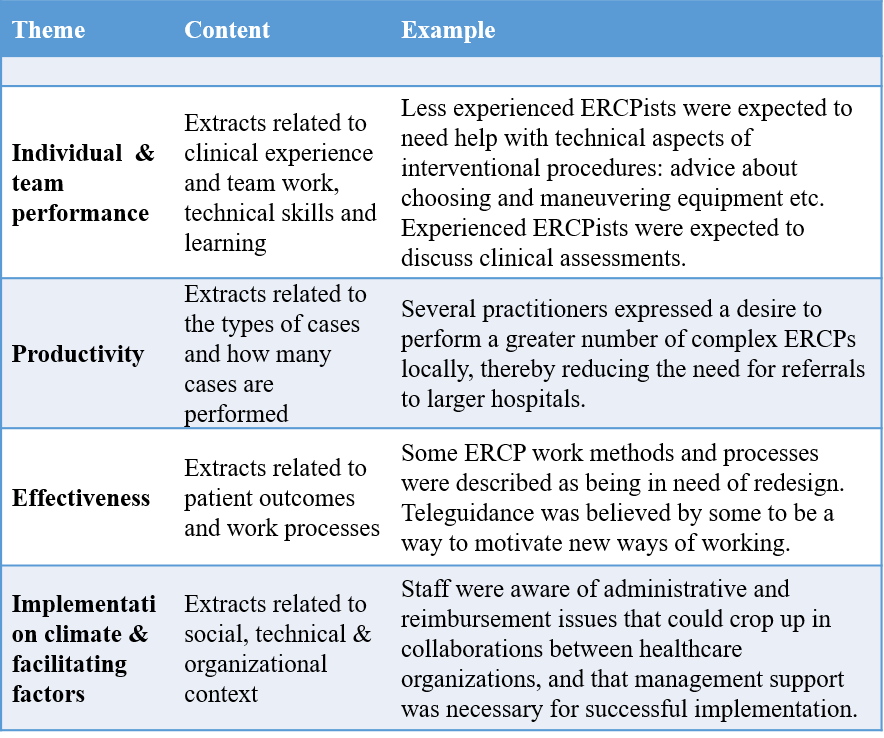

Supplement: Multimedia Appendix 8 [file formative_v5i1e20692_app8.png]

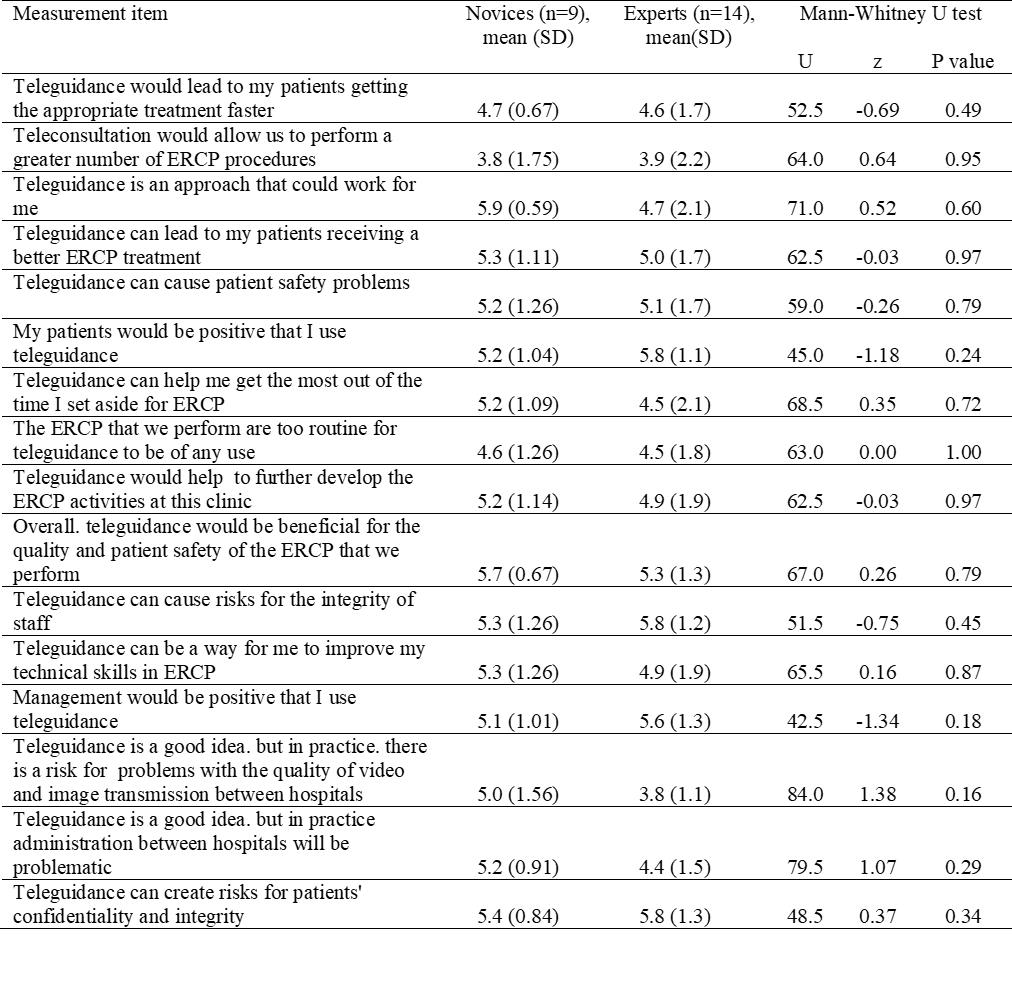

Supplement: Multimedia Appendix 9 [file formative_v5i1e20692_app9.png]
