# Supplementary material for: Telemedicine for Remote Surgical Guidance in Endoscopic Retrograde Cholangiopancreatography: Mixed Methods Study of Practitioner Attitudes
Source: JMIR Form Res. 2021 Jan 11;5(1):e20692. doi: 10.2196/20692 (PMC7834938; doi:10.2196/20692)
Supplement: Multimedia Appendix 3 [file formative_v5i1e20692_app3.docx]

# Consent form: Interviews Teleguidance

You are one of a number of people that we would like to interview and observe in connection with a study on the introduction of distance consultation at ERCP. Participation is voluntary. It is not you as a person and employee who is examined, but our focus is on how you make the work work under realistic conditions.

## About the study

This study aims to provide in-depth knowledge of what activities are carried out at ERCP, and what needs and expectations that relevant personnel have in connection with ERCP. This gives us a basis for being able to measure the benefits of the introduction, and also knowledge of important aspects of your work that need to be taken into account when introducing solutions for remote consultation.

The study is carried out in three stages, where we will now begin in the spring of 2014 with a so-called baseline measurement. Then we will return on two occasions when you have started with a distance consultation.

At this stage, the research focuses mainly on analysis of work by healthcare professionals, not patients or relatives. However, as the study is to some extent carried out at the time of surgery, all affected patients will be asked for approval by a doctor or nurse.

We hope that our presence will not disturb, and would like to emphasize that we are happy to receive comments if something we do is perceived as disturbing or offensive in any way.

During interviews, our conversations will be recorded. We will take notes and even some video recording may occur during observations, to capture details that can be used later in our analyzes.

Your participation is completely voluntary. If you choose to participate, you can, however, cancel your participation at any time.

Your participation is anonymous; you will not be identified in our reports. All interview data will be deidentified, we will not register names or other forms of identity. Data will only be available to the researchers involved in the study.

## Contact information interviews baseline study:

Consent

The research project Remote Consultation at ERCP

I understand the project described above "The research project Remote Consultation at ERCP" and want to participate. I can cancel my participation at any time.

Date: Signature:

Contact information

E-mail address: Telephone number: Serial number:
