# Supplementary material for: Telemedicine for Remote Surgical Guidance in Endoscopic Retrograde Cholangiopancreatography: Mixed Methods Study of Practitioner Attitudes
Source: JMIR Form Res. 2021 Jan 11;5(1):e20692. doi: 10.2196/20692 (PMC7834938; doi:10.2196/20692)
Supplement: Multimedia Appendix 4 [file formative_v5i1e20692_app4.docx]

# Invitation to participate in the questionnaire "Teleguidance in ERCP”

This questionnaire is part of the project "Teleguidance in ERCP ", which is run from Karolinska University Hospital. The survey contain 16 questions and will take approximately 5 minutes to fill out.

Remote ERCP means that an experienced ERCPist is available via video conferencing equipment and can thus participate in the examination by taking a look at the radiology image, the endoscopy image and a picture of the operating room.

First, we want you to read the brief information about remote consulting. Then you rate to what extent you agree with a number of statements about remote consultation. The questions are based on the Technology Acceptance Model (TAM), which is a validated model used to investigate how potential users perceive new technology.

The purpose of the survey is to take note of your views on what is important to consider for remote consultation to work in more hospitals. The results from this survey will be used in the development project for improvements of remote consultation, and will be used in research on the factors that affect the introduction and use of telemedicine. We will handle all information confidentially and no potentially identifying information will appear in publications or other dissemination. The data collected will be used only for research and reviewed and analyzed only by the researchers themselves.

NOTE! We would like you to answer regardless of whether you have experience or knowledge of remote consulting before or not.

Your opinion is important and your participation is highly valued! Thank you in advance for your participation!

## Information about remote consultation

Remote consultation at ERCP is a telemedicine solution that makes it possible to participate in the examination at a distance via video link. What is required is that both parties have telemedicine equipment and have connected to each other by "making" a video call.

In principle, the same activities are carried out as usual at ERCP, with the difference that you first connect the telemedicine equipment to the network socket, endoscopy image and X-ray image, and dial the number for the remote consultation.

By taking part in endoscopy images and X-rays from the operating room, it will be possible for an ERCPist at Karolinska Huddinge to be available at ERCP examinations that are taking place elsewhere.

In the remote consultation, video conferencing equipment is used to communicate and send three different images in real time. The ERCPist at Huddinge can then see two of these three images in parallel: the arrangement in the operating room, X-ray image or endoscopy image. The selection of transmitted image streams is handled via the video system on the page where the procedure is performed.

# Support

Training of MT technicians will be carried out before commissioning so that users can receive local user support.
